# Supplementary material for: Causal Relationship between Aging and Anorexia Nervosa: A White-Matter-Microstructure-Mediated Mendelian Randomization Analysis
Source: Biomedicines. 2024 Aug 16;12(8):1874. doi: 10.3390/biomedicines12081874 (PMC11351342; doi:10.3390/biomedicines12081874)
Supplement: Supplementary file 1 [file biomedicines-12-01874-s001.zip › Supplementary Figure/Figure S4/Singe SNP plot of MR analysis for TL to ubm-b-2086.pdf]

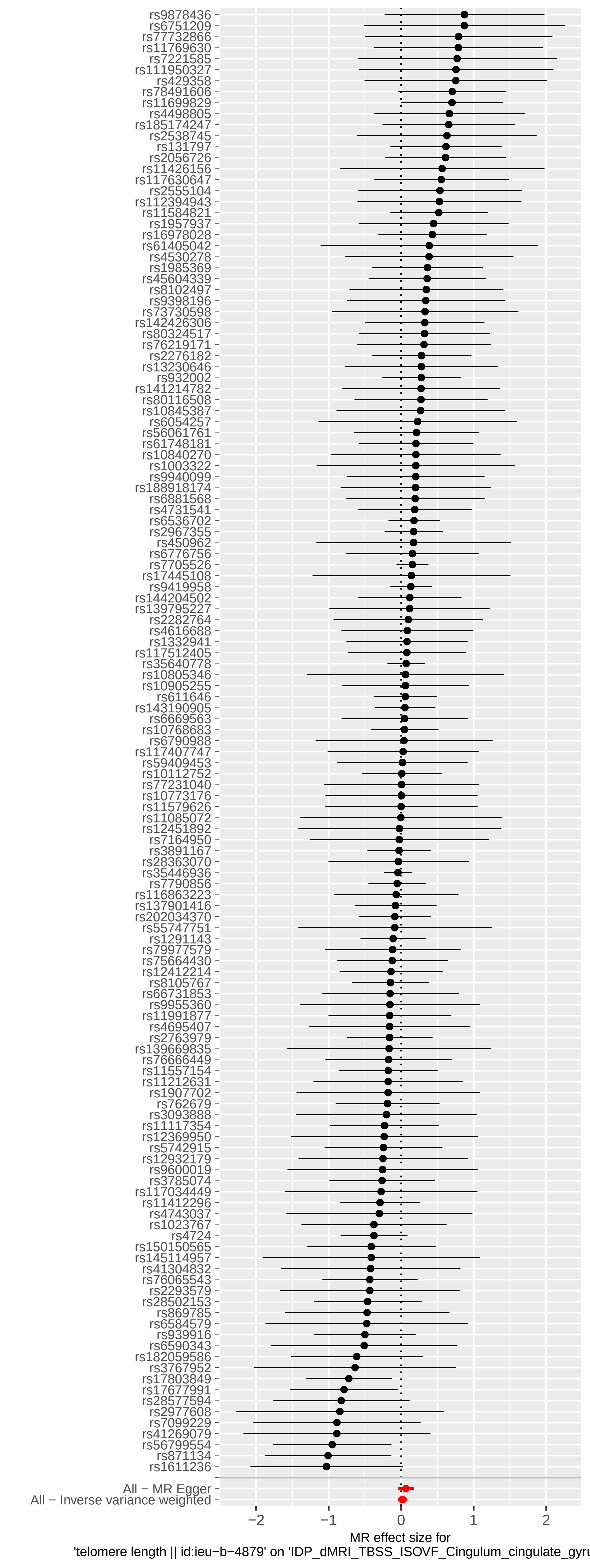
rs9878436
rs6751209
rs77732866
rs11769630
rs7221585
rs111950327
rs429358
rs78491606
rs11699829
rs4498805
rs185174247
rs2538745
rs131797
rs2056726
rs11426156
rs117630647
rs2555104
rs112394943
rs11584821
rs1957937
rs16978028
rs61405042
rs4530278
rs1985369
rs45604339
rs8102497
rs9398196
rs73730598
rs142426306
rs80324517
rs76219171
rs2276182
rs13230646
rs932002
rs141214782
rs80116508
rs10845387
rs6054257
rs56061761
rs61748181
rs10840270
rs1003322
rs9940099
rs188918174
rs6881568
rs4731541
rs6536702
rs2967355
rs450962
rs6776756
rs7705526
rs17445108
rs9419958
rs144204502
rs139795227
rs2282764
rs4616688
rs1332941
rs117512405
rs35640778
rs10805346
rs10905255
rs611646
rs143190905
rs6669563
rs10768683
rs6790988
rs117407747
rs59409453
rs10112752
rs77231040
rs10773176
rs11579626
rs11085072
rs12451892
rs7164950
rs3891167
rs28363070
rs35446936
rs7790856
rs116863223
rs137901416
rs202034370
rs55747751
rs1291143
rs79977579
rs75664430
rs12412214
rs8105767
rs66731853
rs9955360
rs11991877
rs4695407
rs2763979
rs139669835
rs76666449
rs11557154
rs11212631
rs1907702
rs762679
rs3093888
rs11117354
rs12369950
rs5742915
rs12932179
rs9600019
rs3785074
rs117034449
rs11412296
rs4743037
rs1023767
rs4724
rs150150565
rs145114957
rs41304832
rs76065543
rs2293579
rs28502153
rs869785
rs6584579
rs939916
rs6590343
rs182059586
rs3767952
rs17803849
rs17677991
rs28577594
rs2977608
rs7099229
rs41269079
rs56799554
rs871134
rs1611236
All − MR Egger
All − Inverse variance weighted
−2
−1
0
1
2
MR effect size for
'telomere length || id:ieu−b−4879' on 'IDP_dMRI_TBSS_ISOVF_Cingulum_cingulate_gyru
